# Heterofunctional Cationic Polyester Dendrimers as Potent Nonviral Vectors for siRNA Delivery

**DOI:** 10.3390/pharmaceutics17111476

**Published:** 2025-11-16

**Authors:** Arunika Singh, Ángel Buendía, Irene Rodríguez-Clemente, Natalia Sanz del Olmo, Valentín Ceña, Michael Malkoch

**Affiliations:** 1 Division of Coating Technology, Fiber and Polymer Technology, KTH, Teknikringen 48, SE-10044 Stockholm, Sweden; arunika@kth.se (A.S.); natalia.sanzo@uah.es (N.S.d.O.); 2 Unidad Asociada Neurodeath, Institute of Molecular Nanoscience, INAMOL, Universidad de Castilla-La Mancha, 02006 Albacete, Spain; angelbuendiab23@gmail.com (Á.B.); irene.rclemente@uclm.es (I.R.-C.); valentin.cena@gmail.com (V.C.); 3 CIBERNED, Instituto de Salud Carlos III, 28031 Madrid, Spain; 4 Department of Organic and Inorganic Chemistry, Faculty of Sciences, Research Institute in Chemistry “Andrés M. Del Río” (IQAR), University of Alcala, 28805 Madrid, Spain; 5 Institute “Ramón y Cajal” for Health Research (IRYCIS), 28034 Madrid, Spain

**Keywords:** siRNA delivery, cationic polyester dendrimers, protein knockdown, glioblastoma, nuclease protection, dual charge architecture

## Abstract

**Background/Objectives**: Heterofunctional cationic polyester dendrimers derived from a 2-(bromomethyl)-2-(hydroxymethyl)propane-1,3-diol (BHP-diol) based AB_2_C monomer were evaluated as efficient and biodegradable nonviral carriers for siRNA delivery. **Methods**: These dendrimers feature dual internal and external charge architectures, enabling precise control of charge distribution and siRNA interaction strength. **Results**: They achieved complete siRNA complexation at nitrogen-to-phosphate (N/P) ratios of 0.50–2.14 and provided up to 93% RNase protection, outperforming amino-functional scaffolds based on 2,2-bis(methylol)propionic acid (bis-MPA). In human (T98G) and murine (GL261) glioblastoma cells, the dendrimers exhibited minimal cytotoxicity while achieving 52–61% target protein knockdown, a two- to three-fold improvement over conventional polyester dendrimers, and approaching the silencing efficiency of the commercial Interferin^®^ reagent. **Conclusions**: The combination of high complexation efficiency, strong nuclease resistance, and excellent biocompatibility establishes these heterofunctional dendrimers as a new generation of precisely tunable, biodegradable vectors for therapeutic siRNA delivery.

## 1. Introduction

Small interfering RNA (siRNA) therapeutics enable precise and selective gene silencing through sequence-specific mRNA degradation, offering significant potential for targeting oncogenic drivers in cancers such as glioblastoma [1,2]. However, their clinical translation remains limited by the lack of safe and effective delivery systems. Effective siRNA carriers must simultaneously protect siRNA from nuclease-mediated degradation, facilitate cellular uptake, promote endosomal escape, and exhibit low cytotoxicity [3,4]. This challenge is compounded by the inherent instability of naked siRNA, which is rapidly degraded by nucleases and exhibits poor cellular internalization due to its large molecular mass (~13 kDa) and strong anionic charge [5].

Cationic dendrimers have emerged as promising nonviral siRNA delivery systems, combining favorable safety profiles and structural flexibility while avoiding the immunogenicity and insertional mutagenesis risks of viral approaches [3,6]. Their monodisperse architecture and exposed surface functionality enable efficient electrostatic complexation with siRNA, making them promising candidates for therapeutic applications [7]. While amine functional poly(amidoamine) (PAMAM) and carbosilane dendrimers have demonstrated strong siRNA binding and gene silencing capabilities, they are frequently associated with elevated cytotoxicity that limits their biomedical application [6,7,8,9,10]. PAMAM dendrimers are limited by their non-degradable amide backbones and dense surface charge concentration, whereas carbosilane dendrimers, despite improved biocompatibility, often struggle to balance siRNA binding strength with efficient cargo release.

These limitations have encouraged the use of biodegradable polyester dendrimers derived from 2,2-bis(hydroxymethyl)propionic acid (bis-MPA), which contain hydrolytically cleavable ester linkages [9,11]. Stenström et al. developed amino-functional bis-MPA dendrimers from generation 1 to 4 (G1-G4) containing 6 to 48 cationic groups that efficiently complexed and protected siRNA with excellent cytocompatibility in cancer cell lines [12]. However, their homofunctional surface design achieved only moderate gene silencing (~20% protein knockdown in glioma cells) compared to commercial lipofection reagents (~60%), while higher generations (G3 and G4) induced dose-dependent neurotoxicity in primary neurons. These limitations highlight the need for advanced dendrimer designs that enhance siRNA complexation and transfection efficiency while maintaining biocompatibility.

Our group previously established a versatile heterofunctional polyester dendrimer (HFD) platform constructed from BHP-diol, a novel AB_2_C monomer bearing an internal bromide and azide functionality [13,14]. The dendrimers were synthesized through a divergent growth approach using anhydride-mediated esterification, yielding chemically stable and tunable scaffolds with precisely positioned internal and peripheral functionalities. This synthetic strategy enabled independent modulation of internal and external functional group placement, representing a key advance over traditional dendrimers in which charge distribution cannot be independently controlled [12,15]. The resulting platforms demonstrated improved performance in preliminary applications and established the foundation for developing multifunctional dendritic systems with enhanced biological activity.

**Figure 1:**
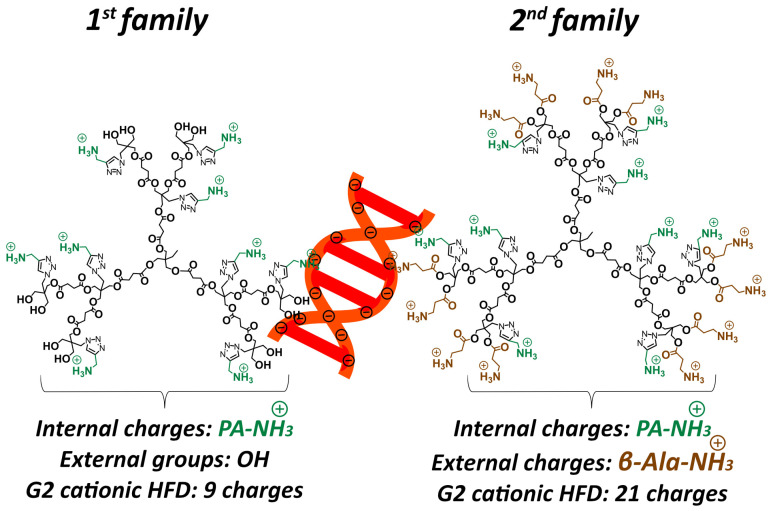
Schematic of second-generation cationic HFDs complexed with siRNA (orange). First family presents internal PA-NH_3_^+^ groups (green), while second family incorporates both internal PA-NH_3_^+^ and external β-Ala-NH_3_^+^ groups (brown).

Building on this platform, we recently applied this architecture to design cationic HFDs with a novel dual-charge configuration, incorporating both internal ammonium groups positioned within the dendrimer backbone and external cationic groups at the dendrimer periphery [15]. In antibacterial applications, this dual-charge design substantially outperformed conventional single-charge architectures, enhancing efficacy while maintaining excellent cytocompatibility. This finding suggests that the spatial localization of charge plays a decisive role in biological performance. The success of this dual-charge approach in antimicrobial applications prompted us to hypothesize that similar architectures could improve siRNA complexation and delivery by optimizing the balance between siRNA binding strength, nuclease protection, and efficient intracellular release, while maintaining the favorable biodegradability of polyester scaffolds. As shown in Figure 1, the two dendrimer families differ fundamentally in their charge architecture. The first family contains only internal propargyl amine cationic groups (PA-NH_3_^+^) positioned within the dendrimer backbone, with neutral hydroxyl groups (OH) at the external termini, yielding a total of 9 cationic charges for the G2 HFD. In contrast, the second family incorporates both internal PA-NH_3_^+^ groups and external β-alanine cationic groups (β-Ala-NH_3_^+^) at the dendrimer periphery, resulting in 21 total cationic charges for the G2 dendrimer (9 internal + 12 external).

In this study, we evaluated the hypothesis of improved siRNA complexation and delivery by assessing the two HFD families with different charge distribution patterns in human (T98G) glioblastoma cells: (1) first family presenting only internal PA-NH_3_^+^ cationic groups, and (2) second family incorporating both internal PA-NH_3_^+^ and peripheral β-Ala-NH_3_^+^ cationic functionalities [15]. Additionally, within the 2nd family we developed a lipophilic dendritic variant by grafting peripheral stearic acid chains to enhance membrane interactions and cellular internalization. Our findings demonstrate that these cationic HFDs achieve high siRNA complexation efficiency and nuclease protection while delivering effective gene silencing with excellent cytocompatibility, establishing them as promising alternatives to traditional dendrimer platforms.

## 2. Materials and Methods

### 2.1. Synthetic Protocols

Heterofunctional cationic polyester dendrimers were synthesized according to previously established protocols [15]. Detailed synthesis procedures and characterization data for the stearic acid-modified dendrimers, are provided in the Supporting Information.

### 2.2. Nuclear Magnetic Resonance (NMR) Spectroscopy

NMR spectroscopy was conducted using a Bruker AM spectrometer (Billerica, Massachusetts, USA) operating at 400 MHz for ^1^H NMR and 101 MHz for ^13^C NMR. ^1^H NMR spectra were acquired using 16 scans with a spectral width of 20 ppm and a relaxation delay of 1 s. The spectrometer employed automated lock and shimming procedures. ^13^C NMR experiments were performed with 256 to 1024 scans depending on the sample concentration and required signal to noise ratio, utilizing a spectral width of 240 ppm and a relaxation delay of 2 s. All NMR data were processed and analyzed using MestreNova software (version 14.2.0-26256, Mestrelab Research S.L., Santiago de Compostela, Galicia, 2020).

### 2.3. Matrix-Assisted Laser Desorption/Ionization Time-of-Flight (MALDI-TOF) Mass Spectrometry

MALDI-TOF analysis was performed using a Bruker UltrafleXtreme MALDI-TOF mass spectrometer (Bruker Daltonics, Bremen, Germany) equipped with a SmartbeamII laser operating at 355 nm in positive ion mode. External mass calibration was conducted using SpheriCal^TM^ calibrants (Polymer Factory Sweden AB, Stockholm, Sweden) to ensure accurate mass determination. Data acquisition and spectral analysis were performed using FlexControl and FlexAnalysis software (Version 3.4, Bruker Daltonics). Matrix solution was prepared by dissolving trans-2-[3-(4-tert-Butylphenyl)-2-methyl-2-propenylidene]malononitrile (DCTB) in tetrahydrofuran (THF) at a concentration of 20 mg mL^−1^. MALDI sample preparation was performed by sequential deposition of 1 μL of analyte solution (1 mg mL^−1^) followed by 2 μL of matrix solution on an MPT 284 Target ground steel TF Target (Bruker Daltonics). The prepared MALDI spots were allowed to dry at room temperature. Mass spectra were acquired in reflector mode with an acceleration voltage of 25 kV and a reflector voltage of 26.3 kV. The laser intensity was optimized between 50 and 100% to achieve optimal signal intensity and spectral resolution for each sample.

### 2.4. Size Exclusion Chromatography (SEC)

Molecular weight determination and polydispersity analysis were performed using SEC on a TOSOH EcoSEC HLC-8320GPC system equipped with an EcoSEC RI detector (Shunan City, Japan). The chromatographic separation was achieved using three PSS PFG columns (5 μm particle size; Microguard, 100 Å, and 300 Å pore sizes) connected in series (PSS GmbH, Mainz, Germany), providing a molecular weight resolving range of 300–100,000 Da. The measurements were performed in mobile phase consisting of dimethylformamide (DMF) and 0.01 M lithium bromide (LiBr) at 35 °C. Sample concentrations were prepared in the range of 2–3 mg mL^−1^ for the post-functionalized dendrimers. External calibration was performed using narrow polydispersity poly(methyl methacrylate) (PMMA) standards (PSS GmbH). Toluene was employed as an internal standard to correct for minor flow rate fluctuations. Data acquisition and processing were conducted using WinGPC Unity software (Version 7.2). The obtained chromatograms were subsequently normalized and plotted using Origin software (Version 9.1.0 Sr1).

### 2.5. Cell Culture

Human and murine glioblastoma cell lines were utilized in this study. The T98G human glioblastoma cell line was obtained from the American Type Culture Collection (ATCC, Manassas, VA, USA), and the GL261 murine glioma cell line was acquired from the Leibniz-Institut DSMZ (Braunschweig, Germany). All cell lines were cultured according to the respective provider’s protocols. Briefly, cells were maintained in Dulbecco’s Modified Eagle Medium (DMEM) supplemented with 10% heat-inactivated fetal bovine serum (FBS), 1–2% L-glutamine, and 1% penicillin/streptomycin antibiotic mixture. Cultures were incubated at 37 °C in a humidified atmosphere containing 5% CO_2_ for T98G cell lines and 10% CO_2_ for GL261 cells.

### 2.6. Agarose Gel Retardation

Agarose gel electrophoresis was performed to evaluate siRNA complexation efficiency following established protocols [12,16]. Briefly, nanoparticle/siRNA complexes were prepared by mixing 100 nM siRNA with increasing concentrations of dendrimers (0.1–10 μM) to achieve the desired nitrogen to phosphate (N/P) molar ratios. The mixtures were incubated at room temperature for 30 min with gentle agitation to facilitate complex formation. Subsequently, samples were mixed with 2 μL 10× loading buffer, and 20 μL of each complexation mixture was loaded onto a 1.8% (*w*/*v*) agarose gel containing RedSafe™ nucleic acid staining solution (1:10,000 dilution). Electrophoresis was conducted in 1× TAE buffer (40 mM Tris-acetate, 1 mM EDTA, pH 8.3) at 60 V for 10 min. Post-electrophoresis, gels were visualized under ultraviolet illumination using a gel documentation system (Vilber, Marne-la-Vallée, France), and quantitative analysis was performed using ImageJ version 1.54p [17].

### 2.7. RNase Protection Assay of siRNA-Dendrimer Complexes

The protective capability of dendrimer-siRNA complexes against RNase-mediated degradation was evaluated following established methodologies [12,18]. Dendrimer-siRNA complexes were prepared by incubating nanoparticles with 100 nM siRNA at specified N/P ratios for 30 min at room temperature to facilitate complex formation. Subsequently, RNase A (0.25% *w*/*v*; Sigma-Aldrich, Barcelona, Spain) was added to the mixtures, followed by incubation at 37 °C for 30 min. RNase activity was terminated by immediate cooling to 4 °C for 15 min. Heparin (0.5 USP units) was then introduced to displace siRNA from the dendrimer complexes, with the mixtures maintained at 4 °C for an additional 20 min to ensure complete siRNA release while preventing RNase reactivation [19]. The resulting samples were subjected to electrophoresis on a 1.2% (*w*/*v*) agarose gel containing ethidium bromide (1:10,000 dilution) in 1× TAE buffer. Electrophoresis was conducted at 60 V for 10 min. Post-electrophoresis, gels were visualized under UV illumination using a gel documentation system (Vilber, Marne-la-Vallée, France). Fluorescent bands corresponding to siRNA were digitized and analyzed using ImageJ version 1.54p to assess the integrity and protection efficiency of the siRNA [17].

### 2.8. siRNA Transfection and Protein Knockdown Analysis

Cells were treated with dendriplexes prepared by incubating dendrimers (1 μM) with either scrambled or gene-specific siRNAs (25–100 nM) in serum-free medium for 30 min at room temperature [12,20]. siRNAs (Sigma-Aldrich, Barcelona, Spain) were designed to specifically target mRNAs encoding two proteins involved in survival and proliferation of glioblastoma cells such as p42-Mitogen-Activated Protein Kinases (p42-MAPK) and Ras homolog enriched in brain (Rheb). After 72 h treatment, the medium was washed twice and the cells were lysed, followed by determination of protein concentrations by bicinchoninic acid (BCA) protein assay (Pierce Biotechnology, Rockford, IL, USA). Proteins (20–30 μg) were loaded onto the gel, separated in a 12–15% SDS-PAGE and transferred to PVDF membranes [12,21]. The primary antibodies to correct for protein loading included anti-p42-MAPK (1:500) (Cell Signalling Technology, Leiden, The Netherlands), anti-Rheb (1:500) (R&D Systems, Minneapolis, MN, USA), and anti-β-actin (1:2000) (Sigma Chemical Co., St. Louis, MO, USA). The protein bands were visualized using an enhanced chemiluminescence (ECL) detection system (Millipore, Burlington, MA, USA), and densitometric quantification was performed using ImageJ version 1.54p [17].

### 2.9. Cytotoxicity Studies

Cell viability was assessed using the lactate dehydrogenase (LDH) release assay as a marker of membrane integrity and cell death [12,22]. T98G and GL261 cells were seeded in 24-well plates at a concentration of 1 × 10^5^ and 2 × 10^5^ cells/mL, respectively, and treated with dendrimer concentrations ranging from 0.1 to 10 μM for 72 h. The culture supernatants were collected, and the adherent cells were washed with phosphate-buffered saline (PBS) and lysed using 0.9% (*v*/*v*) Triton X-100. The LDH activity in supernatants and lysates was measured spectrophotometrically at 490 nm using the Cytotox 96 kit (Promega, Madrid, Spain). Toxicity was expressed as the percentage of LDH released relative to the total LDH content of the cells, as previously described [12,22].

### 2.10. Statistical Analysis

The nonparametric variance analysis (Kruskal–Wallis) followed by Dunn’s test was used to evaluate statistical differences between groups. *p* < 0.05 was considered statistically significant. Statistical analyses were performed using GraphPad software 5 (GraphPad Software, Boston, MA, USA).

## 3. Results and Discussion

**Scheme 1:**
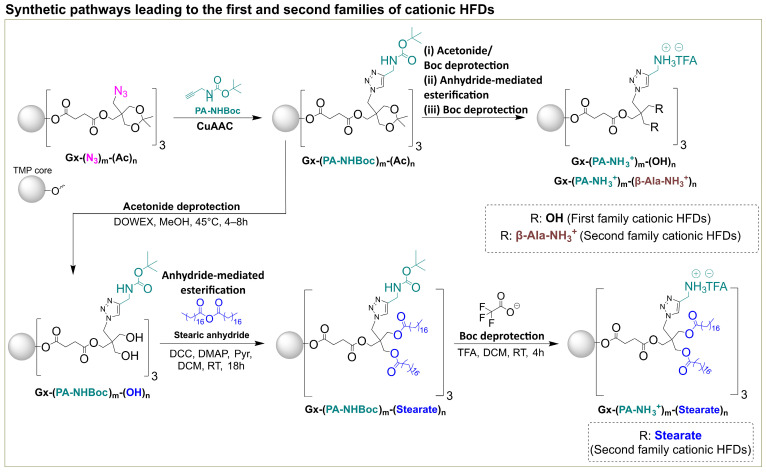
Synthetic pathways leading to the first and second families of cationic HFDs [14,15]. The scheme illustrates the complete synthetic strategy for generating both dendrimer families from an azide-based HFD (Gx-(N_3_)_m_-(Ac)_n_), where x represents the dendrimer generation. (i) Post-functionalization via click chemistry to incorporate Boc protected internal propargyl amine functionalities (PA-NHBoc)_m_. (ii) Subsequent TFA deprotection yields the first family HFDs containing only internal cationic ammonium groups (Gx-(PA-NH_3_^+^)_m_-(OH)_n_). (iii) For the second family, an additional post-functionalization step with β-alanine introduces external cationic groups (β-Ala-NH_3_^+^)_n_, yielding the dual-charge architecture (Gx-(PA-NH_3_^+^)_m_-(β-Ala-NH_3_^+^)_n_). The lipophilic variant (Gx-(PA-NH_3_^+^)_m_-(Stearate)_n_) was synthesized by anhydride-mediated esterification with stearic anhydride prior to final Boc deprotection. Abbreviations: Trimethylolpropane (TMP), N,N′-dicyclohexylcarbodiimide (DCC), 4-dimethylaminopyridine (DMAP), Trifluoroacetic acid (TFA); x, generation number; m and n, numbers of internal and external functionalities.

Two families of cationic HFDs were synthesized following established protocols (Scheme 1) [15]. The first family contained only internal PA-NH_3_^+^ charges, while the second family incorporated both internal PA-NH_3_^+^ and external β-Ala-NH_3_^+^ groups, providing enhanced charge density and accessibility. To explore the effect of lipophilic modification on cellular interactions, a novel derivative, G2-(PA-NH_3_^+^)_9_-(Stearate)_12_, was prepared by anhydride mediated esterification of the tert-butoxycarbonyl (Boc)-protected hydroxyl precursor with stearic anhydride, followed by trifluoroacetic acid (TFA) deprotection to yield the fully deprotected dendrimer as a TFA salt in 98% yield (Scheme 1). The successful incorporation of twelve stearic acid chains while maintaining nine internal cationic charges was confirmed by comprehensive spectroscopic analysis. ^1^H NMR revealed characteristic aliphatic proton signals between 0.84 and 1.28 ppm corresponding to the stearic acid chains (Figure S4), while MALDI-TOF MS showed the expected molecular weight peak at *m*/*z* 6048.49 Da ([M+Na]^+^, Figure S7), confirming complete substitution and structural integrity. All dendrimers were characterized by ^1^H NMR, ^13^C NMR, MALDI-TOF mass spectrometry, and SEC, confirming their expected structures and molecular weights (Figures S1–S7).

**Figure 2:**
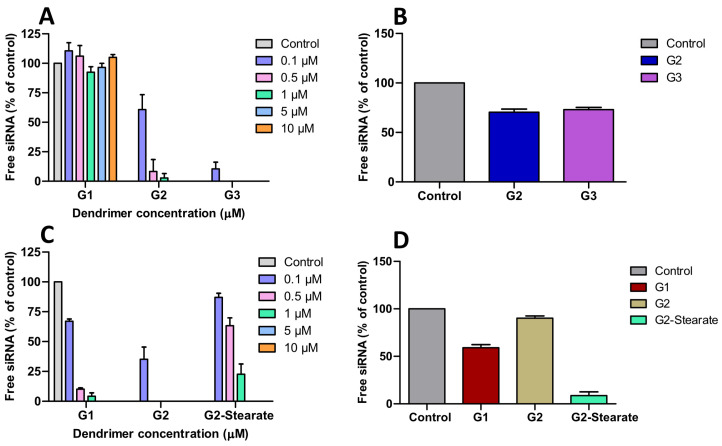
(**A**,**C**) Agarose gel retardation assay of siRNA (100 nM) complexed with the first and second family cationic HFDs at indicated N/P ratios, resolved on 1.8% agarose gels. (**B**,**D**) RNase protection assay of siRNA complexes formed with the first (G2, G3) and second family (G1, G2, G2-Stearate) dendrimers at N/P = 2.14, followed by RNAse treatment and heparin displacement. Free siRNA refers, in (**A**,**C**), to the amount of intact siRNA not bound by the dendrimers. In the case of (**B**,**D**), it represents the amount of intact siRNA recovered from the dendrimer after exposure of the dendriplex to RNase and subsequent inactivation of the enzyme. Control, in (**B**,**D**), represents the amount of siRNA added to the dendrimers at the beginning of the experiment. siRNA was quantified by densitometric analysis using ImageJ version 1.54p as indicated in Methods. Data represent mean ± s.e.m. (*n* = 3–4 independent experiments).

Agarose gel retardation assays (100 nM siRNA) revealed significant architectural advantages of cationic HFDs over bis-MPA dendrimers. The first family displayed a generation-dependent trend towards siRNA binding at the indicated nitrogen (ammonium cationic groups) to phosphate (siRNA) ratios: G1 showed negligible binding (N/P = 7.14), G2 achieved complete binding at N/P of 2.14, and G3 at N/P of 0.50 (Figure 2A). In contrast, the second-family dendrimers exhibited markedly superior efficiency, with G1 binding at N/P of 1.07 (a seven-fold improvement over its first family counterpart) and G2 matching the performance of G3 from first-family despite being a lower generation dendrimer but possessing equivalent cationic charge density (21 charges) (Figure 2C and Figure S8). The improved siRNA binding reflects the dual benefit of increased charge number and enhanced accessibility through externally positioned cationic groups, demonstrating that charge distribution and accessibility are critical determinants of dendrimer-siRNA interactions. The stearate modified derivative maintained strong siRNA binding despite the reduced external surface charge (Figure 2C and Figure S8). Compared to bis-MPA dendrimers requiring N/P ratios of 1.5–3 (G2–G4) and impractically high ratios for G1 (>19), these dual-charge scaffolds deliver three- to six-fold enhancement in siRNA complexation efficiency [12]. This improvement enables even low-generation dendrimers to efficiently bind siRNA, overcoming a key limitation of traditional polyester dendrimers, where effective binding is typically restricted to higher generations. This enhancement is consistent with our previous antibacterial studies, where dendrimers containing both internal and external charges demonstrated superior bacteriostatic and bactericidal performance compared to the internally charged analogs [15]. RNase protection assays further confirmed the stability advantages of these HFD-siRNA complexes.

After RNase A treatment (30 min) followed by heparin displacement, the G2 and G3 dendrimers from first family preserved approximately 75% of intact siRNA (Figure 2B), while the G1 and G2 dendrimers from second family afforded 63% and 93% siRNA protection against nucleases, respectively, (Figure 2D and Figure S9). The high RNase protection achieved by G2-(PA-NH_3_^+^)_9_-(β-Ala-NH_3_^+^)_12_ represents a 1.2-fold improvement over the ~80% protection reported for G2 bis-MPA scaffold, demonstrating superior nuclease resistance through optimized charge distribution [12]. The reduced performance of G2-(PA-NH_3_^+^)_9_-(Stearate)_12_ may be attributed either to excessively strong siRNA binding that hinders its release upon heparin treatment or to insufficient protection against degradation [23] (Figure 2D and Figure S9).

**Figure 3:**
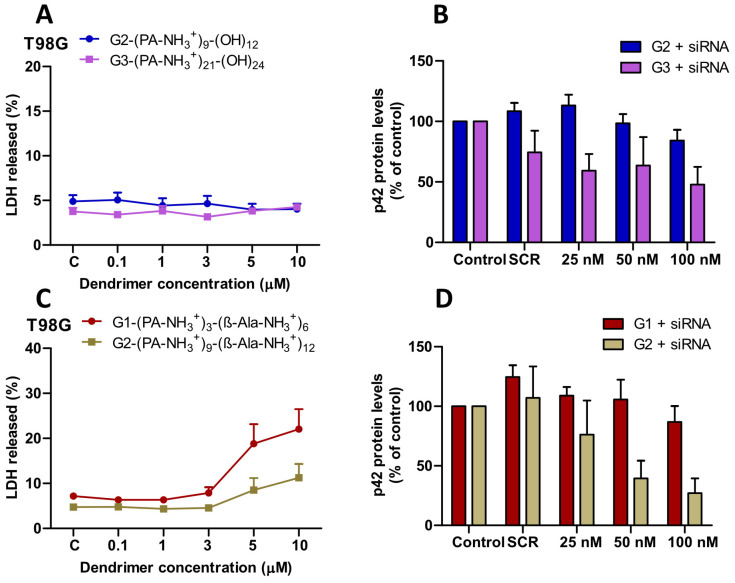
(**A**,**C**) Cytotoxicity of the first family (G2, G3) and second family (G1, G2) cationic HFDs in T98G cells following 72 h exposure at the indicated concentrations (0.1–10 μM). Data are presented as mean ± s.e.m. (*n* = 3–4 independent experiments). (**B**,**D**) Protein knockdown in T98G cells transfected with dendrimer-siRNA complexes (25–100 nM siRNA) targeting p42-MAPK. Protein levels were quantified after 72 h by Western blot analysis. Data represent mean ± s.e.m (*n* = 3–4 independent experiments).

Lactate dehydrogenase (LDH) release assays in T98G glioblastoma cells demonstrated excellent biocompatibility of both dendritic families. The first family (G2, G3) maintained approximately 5% LDH release across all concentrations tested up to 10 μM (Figure 3A). The second family exhibited a similar low toxicity profile up to 3 μM, followed by a concentration-dependent increase until 10 μM, reaching ~23% release for the G1 and ~12% for the G2 dendrimer (Figure 3C). Consistent results were observed in GL261 murine glioma cells, where the second family also showed minimal toxicity at all tested concentrations (Figure S10). The cytotoxicity profile of these dendrimers significantly outperformed amino-functional bis-MPA dendrimers, where the G3 and G4 dendrimers exhibited dose-dependent neurotoxicity at concentrations as low as 0.1–1 μM [12]. The superior safety profile of cationic HFDs likely arises from their distributed charge architecture, which reduces local charge density compared to the surface-concentrated charges of bis-MPA scaffolds.

Both dendrimer families achieved significant, dose-dependent protein level reduction in T98G cells through siRNA-mediated inhibition of target genes. For p42-MAPK targeting, the G3 dendrimer from the first family mediated protein knockdown from ~27% at 25 nM to ~38% at 100 nM siRNA (Figure 3B), whereas G2 achieved only ~16% knockdown at the highest concentration. In the second family, the G2 dendrimer performed strongly, achieving ~45% knockdown at 50 nM and ~62% at 100 nM, while G1 remained inactive (Figure 3D).

For Rheb targeting, the G2 dendrimer from the first family mediated protein knockdown from ~20% at 25 nM to ~51% at 100 nM siRNA (Figure S11), whereas G3 achieved only ~19% knockdown despite stronger siRNA binding. This outcome indicates that excessively tight siRNA binding can hinder in vitro release, highlighting the need to balance siRNA protection with bioavailability [23]. In the second family, only the G2 dendrimer achieved 15–24% Rheb knockdown at higher siRNA concentrations, while G1 showed no activity (Figure S11).

Importantly, the collected results from the protein knockdown studies represent two- to three-fold improvements over bis-MPA scaffolds (~20% knockdown) and approach the ~60% silencing obtained with commercial Interferin^®^ transfection reagent [12].

The enhanced efficacy of cationic HFDs reflects several architectural advantages, including improved binding at lower N/P ratios (0.50–2.14 versus 1.5–3 for bis-MPA dendrimers), stronger RNase protection (93% versus ~80%), and distributed charge effects that likely promote endosomal escape through enhanced proton sponge mechanisms [24]. The superior performance of the G2 dendrimers from both families further highlights the importance of achieving an optimal binding affinity that ensures siRNA stability while enabling efficient functional release.

## 4. Conclusions

Heterofunctional cationic polyester dendrimers represent a significant advancement in siRNA delivery technology. By strategically integrating internal and external cationic groups, these systems overcome the key limitations of traditional bis-MPA dendrimers by enhancing siRNA binding efficiency, nuclease protection, and biocompatibility. Second-generation dendrimers from both families demonstrated optimal performance, achieving efficient siRNA complexation at low N/P ratios, strong nuclease protection (up to 93%), and effective gene silencing (51–62%) with minimal cytotoxicity. Notably, their silencing efficiency approaches that obtained with the commercial Interferin^®^ transfection reagent (~60%), emphasizing the competitiveness of these biodegradable, nonviral carriers. Importantly, their superior performance compared to the higher-generation (G3) dendrimer of the first family highlights the critical role of balancing siRNA binding affinity with cargo release, a design principle that distinguishes these scaffolds from conventional dendrimers, where higher generations typically perform better. The dual-charge architecture provides precise control over this balance, resulting in carriers that both protect siRNA during delivery and facilitate efficient release for gene silencing. Collectively, these findings establish cationic HFDs as superior siRNA delivery platforms compared to bis-MPA scaffolds, directly overcoming the efficacy limitations that have restricted their in vitro performance. The stearate-modified variant G2-(PA-NH_3_^+^)_9_-(Stearate)_12_ represents an exploratory strategy to enhance dendrimer-membrane interactions via lipophilic surface modification. Stearic acid residues may serve as hydrophobic anchors, facilitating dendrimer insertion into cellular membranes and promoting uptake through membrane-mediated endocytosis. However, our findings reveal a trade-off: while this variant retains strong siRNA binding, it exhibits reduced RNase protection, suggesting that enhanced membrane affinity may lead to premature siRNA release before intracellular delivery. Optimizing stearate chain density and spatial arrangement will be critical to balance membrane interaction with effective nuclease protection. These lipophilic dendrimers show potential for improving cellular uptake, particularly in tissue-targeted gene delivery applications.

Future work will focus on new dendrimer designs with controlled spatial charge manipulation, enabling further tuning of siRNA binding strength, endosomal escape, and intracellular release, as well as in vivo evaluation and incorporation of targeting ligands to enhance tissue selectivity in glioblastoma therapy.

## Data Availability

The original contributions presented in this study are included in the article/Supplementary Materials. Further inquiries can be directed to the corresponding author.

## Supplementary Materials

The following supporting information can be downloaded at: https://www.mdpi.com/article/10.3390/pharmaceutics17111476/s1, Figure S1: ^1^H and ^13^C NMR spectra of Stearic anhydride in CDCl_3_; Figure S2: ^1^H and ^13^C NMR spectra of G2-(PA-NHBoc)_9_-(Stearate)_12_ in CDCl_3_; Figure S3: DOSY spectra of G2-(PA-NHBoc)_9_-(Stearate)_12_ in CDCl_3_; Figure S4: ^1^H and ^13^C NMR spectra of G2-(PA-NH_3_^+^)_9_-(Stearate)_12_ in CDCl_3_; Figure S5: SEC of G2-(PA-NHBoc)_9_-(Stearate)_12_; Figure S6: MALDI of G2-(PA-NHBoc)_9_-(Stearate)_12_ in DCTB; Figure S7: MALDI of G2-(PA-NH_3_^+^)_9_-(Stearate)_12_ in DCTB; Figure S8: Agarose gel retardation assay of siRNA (100 nM) complexed with G1-(PA-NH_3_^+^)_3_-(β-Ala-NH_3_^+^)_6_, G2-(PA-NH_3_^+^)_9_-(β-Ala-NH_3_^+^)_12_ and G2-(PA-NH_3_^+^)_9_-(Stearate)_12_ from the 2nd family at concentrations ranging from 100 nM to 10 μM. Gel electrophoresis images showing lane 1 as free siRNA (control), with subsequent lanes corresponding to increasing dendrimer concentrations; Figure S9: RNase protection assay of siRNA (100 nM) by the 2nd family cationic dendrimers at 1 µM (G1-(PA-NH_3_^+^)_3_-(β-Ala-NH_3_^+^)_6_, G2-(PA-NH_3_^+^)_9_-(β-Ala-NH_3_^+^)_12_) and 5 µM (G2-(PA-NH_3_^+^)_9_-(Stearate)_12_). Dendriplexes were treated with RNase A (0.25 mg/mL) for 30 min at 37 °C, followed by heparin displacement and agarose gel electrophoresis. Lane assignments: siRNA control, siRNA + nanoparticle, siRNA + RNase A + heparin, and dendrimer control (nanoparticle + RNase A+ heparin); Figure S10: Cytotoxicity of the 2nd family (G1-(PA-NH_3_^+^)_3_-(β-Ala-NH_3_^+^)_6_, G2-(PA-NH_3_^+^)_9_-(β-Ala-NH_3_^+^)_12_) cationic heterofunctional dendrimers in GL261 cells following 72 h exposure at the indicated concentrations (0.1–10 µM). Data are presented as mean ± s.e.m. (*n* = 3–4 independent experiments); Figure S11: Protein knockdown in T98G cells transfected with dendrimer-siRNA complexes (25–100 nM siRNA) targeting Rheb. Protein levels were quantified after 72 h by Western blot analysis. Data represent mean ± s.e.m (*n* = 3–4 independent experiments).

## Abbreviations

The following abbreviations are used in this manuscript:

Boc	Di-tert-butyl decarbonate
PA-NHBoc	Boc protected propargyl amine
PA-NH_3_^+^	Propargylammonium trifluoroacetate
β-Ala-NH_3_^+^	β-alaninium trifluoroacetate
DCC	N,N′-Dicyclohexylcarbodiimide
DCM	Dichloromethane
DCU	1,3-Dicyclohexyl urea
DCTB	Trans-2-[3-(4-tert-Butylphenyl)-2-methyl-2-propenylidene]malononitrile
DMEM	Dulbecco’s modified Eagle medium
DMF	Dimethylformamide
DMAP	4-(Dimethylamino)pyridine
EtOAc	Ethyl acetate
FBS	Fetal bovine serum
GL261	Murine glioma cells
Hep	Heptane
MALDI-TOF	Matrix-assisted laser desorption ionization time-of-flight
MeOH	Methanol
NMR	Nuclear magnetic resonance
SEC	Size Exclusion Chromatography
TFA	Trifluoroacetic acid
THF	Tetrahydrofuran
T98G	Human glioblastoma multiforme cells
