# Supplementary material for: Heterofunctional Cationic Polyester Dendrimers as Potent Nonviral Vectors for siRNA Delivery
Source: Pharmaceutics. 2025 Nov 16;17(11):1476. doi: 10.3390/pharmaceutics17111476 (PMC12655120; doi:10.3390/pharmaceutics17111476)
Supplement: Supplementary file 1 [file pharmaceutics-17-01476-s001.zip › pharmaceutics-3961040-supplementary.pdf]

Electronic Supporting Information

Heterofunctional Cationic Polyester Dendrimers as Potent Nonviral Vectors for siRNA Delivery

Arunika Singh [1], Ángel Buendía [2,3], Irene Rodríguez-Clemente [2,3], Natalia Sanz del Olmo [1,4,5], Valentín Ceña [2,3] and Michael Malkoch [1,*]

[1] Division of Coating Technology, Fiber and Polymer Technology, KTH, Teknikringen 48,
SE-10044 Stockholm, Sweden; arunika@kth.se (A.S.); natalia.sanzo@uah.es (N.S.d.O.)
[2] Unidad Asociada Neurodeath, Institute of Molecular Nanoscience, INAMOL, Universidad de Castilla-La Mancha, 02006 Albacete, Spain; angelbuendiab23@gmail.com (Á.B.); irene.rclemente@uclm.es (I.R.-C.); valentin.cena@gmail.com (V.C.)
[3] CIBERNED, Instituto de Salud Carlos III, 28031 Madrid, Spain
[4] Department of Organic and Inorganic Chemistry, Faculty of Sciences, University of Alcala, and Research Institute in Chemistry "Andrés M. Del Río" (IQAR), 28805 Madrid, Spain
[5] Institute "Ramón y Cajal" for Health Research (IRYCIS), 28034 Madrid, Spain
* Correspondence: malkoch@kth.se; Tel.: +46-08-790-82-83

Contact info: School of Engineering Sciences in Chemistry, Biotechnology and Health; Department of Fibre and Polymer Technology; Division of Coating Technology Teknikringen 48, SE-10044, Stockholm (malkoch@kth.se) Fax: (+) 46 (0)8 790 82 83.

Table of Contents

General information 3

Synthesis protocols 4

Figures 8

Figure S1. ^{1}H and ^{13}C NMR spectra of Stearic anhydride in $CDCl_3$

Figure S2. ^{1}H and ^{13}C NMR spectra of G2-(PA-NHBoc)$_9$-(Stearate)$_{12}$ in $CDCl_3$

Figure S3. DOSY spectra of G2-(PA-NHBoc)$_9$-(Stearate)$_{12}$ in $CDCl_3$

Figure S4. ^{1}H and ^{13}C NMR spectra of G2-(PA-NH_3^+)$_9$-(Stearate)$_{12}$ in $CDCl_3$

Figure S5. SEC of G2-(PA-NHBoc)$_9$-(Stearate)$_{12}$

Figure S6. MALDI of G2-(PA-NHBoc)$_9$-(Stearate)$_{12}$ in DCTB

Figure S7. MALDI of G2-(PA-NH_3^+)$_9$-(Stearate)$_{12}$ in DCTB

Figure S8. Agarose gel retardation assay of siRNA (100 nM) complexed with G1-(PA-NH_3^+)$_3$-(β-Ala-NH_3^+)$_6$, G2-(PA-NH_3^+)$_9$-(β-Ala-NH_3^+)$_{12}$ and G2-(PA-NH_3^+)$_9$-(Stearate)$_{12}$ from the 2nd family at concentrations ranging from 100 nM to 10 µM. Gel electrophoresis images showing lane 1 as free siRNA (control), with subsequent lanes corresponding to increasing dendrimer concentrations

Figure S9. RNase protection assay of siRNA (100 nM) by the 2nd family cationic dendrimers at 1 µM (G1-(PA-NH_3^+)$_3$-(β-Ala-NH_3^+)$_6$, G2-(PA-NH_3^+)$_9$-(β-Ala-NH_3^+)$_{12}$) and 5 µM (G2-(PA-NH_3^+)$_9$-(Stearate)$_{12}$). Dendriplexes were treated with RNase A (0.25 mg/mL) for 30 minutes at 37°C, followed by heparin displacement and agarose gel electrophoresis. Lane assignments: siRNA control, siRNA + nanoparticle, siRNA + RNase A + heparin, and dendrimer control (nanoparticle + RNase A+ heparin)

Figure S10. Cytotoxicity of the 2nd family (G1-(PA-NH_3^+)$_3$-(β-Ala-NH_3^+)$_6$, G2-(PA-NH_3^+)$_9$-(β-Ala-NH_3^+)$_{12}$) cationic heterofunctional dendrimers in GL261 cells following 72h exposure at the indicated concentrations (0.1-10 µM). Data are presented as mean ± s.e.m. (n= 3-4 independent experiments)

Figure S11. Protein knockdown in T98G cells transfected with dendrimer-siRNA complexes (25-100 nM siRNA) targeting Rheb. Protein levels were quantified after 72 h by Western blot analysis. Data represent mean ± s.e.m (n= 3-4 independent experiments)

References 14

General Information

Abbreviations

Boc	Di-tert-butyl dicarbonate
PA-NHBoc	Boc protected propargyl amine
$PA\text{-}NH_3^+$	Propargylammonium trifluoroacetate
$\beta\text{-}Ala\text{-}NH_3^+$	β-alaninium trifluoroacetate
DCC	N,N'-Dicyclohexylcarbodiimide
DCM	Dichloromethane
DCU	1,3-Dicyclohexyl urea
DCTB	Trans-2-[3-(4-tert-Butylphenyl)-2-methyl-2-propenylidene]malononitrile
DMEM	Dulbecco's modified Eagle medium
DMF	Dimethylformamide
DMAP	4-(Dimethylamino)pyridine
EtOAc	Ethyl acetate
FBS	Fetal bovine serum
GL261	Murine glioma cells
Hep	Heptane
MALDI-TOF	Matrix-assisted laser desorption ionization time-of-flight
MeOH	Methanol
NMR	Nuclear magnetic resonance
SEC	Size Exclusion Chromatography
TFA	Trifluoroacetic acid
THF	Tetrahydrofuran
T98G	Human glioblastoma multiforme cells

Synthesis protocols

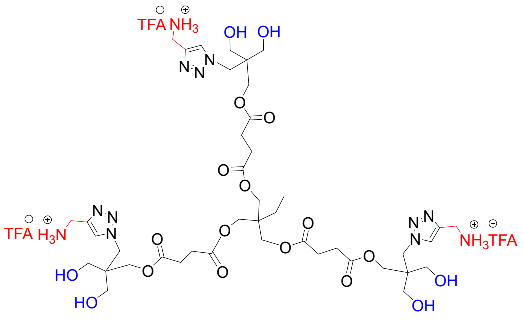

A

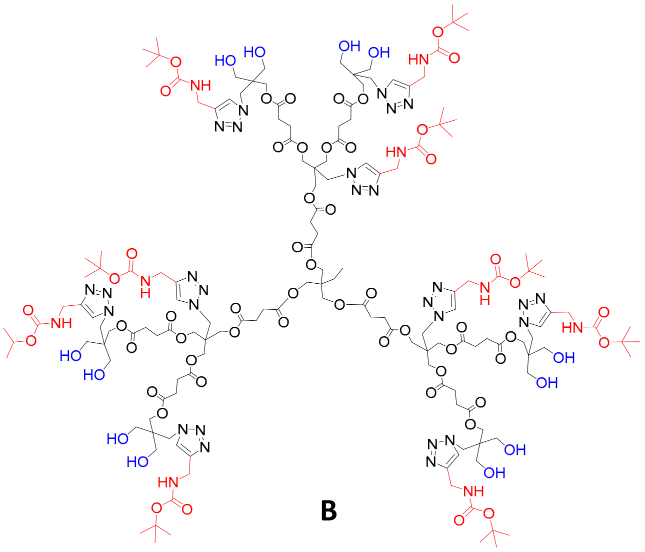

B

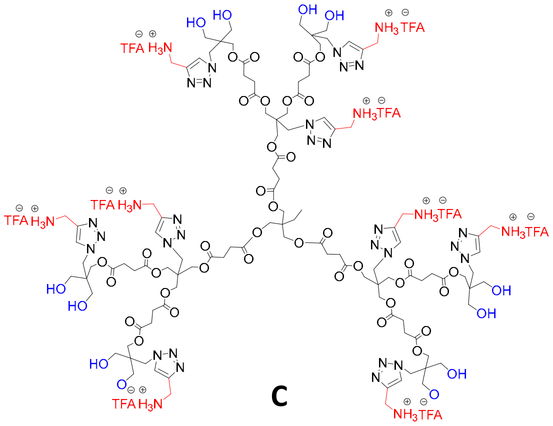

C

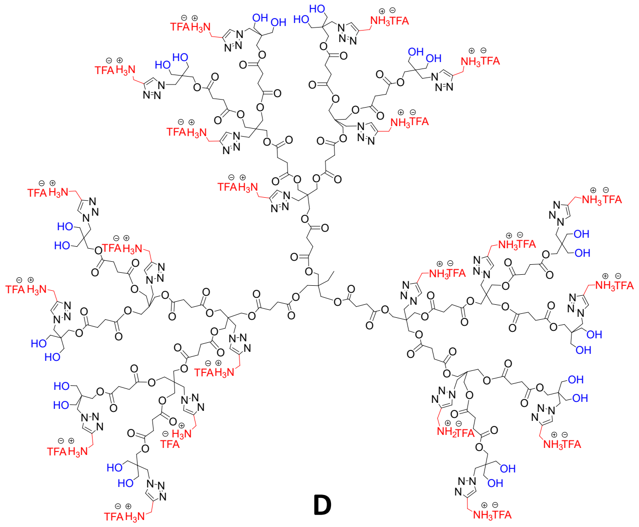

D

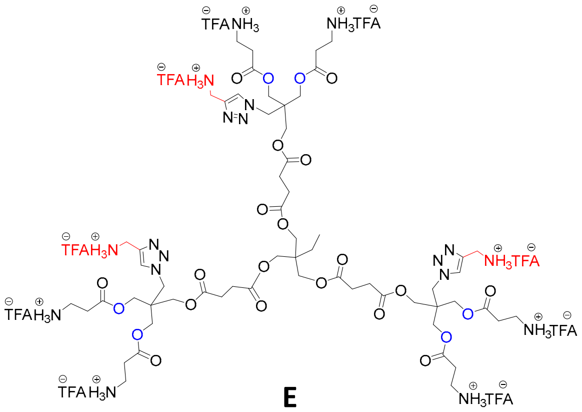

E

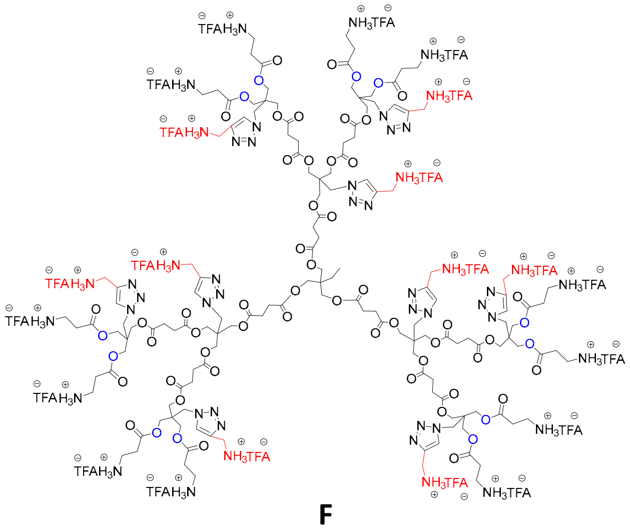

F

The compounds shown above were synthesized according to procedures described in our earlier publication.[1] A) G1-(PA-NH_3^+)$_3$-(OH)$_6$[1] B) G2-(PA-NHBoc)$_9$-(OH)$_{12}$[1] C) G2-(PA-NH_3^+)$_9$-(OH)$_{12}$[1] D) G3-(PA-NH_3^+)$_{21}$-(OH)$_{24}$[1] E) G1-(PA-NH_3^+)$_3$-(β-Ala-NH_3^+)$_6$[1] F) G2-(PA-NH_3^+)$_9$-(β-Ala-NH_3^+)$_{12}$[1]

Stearic anhydride

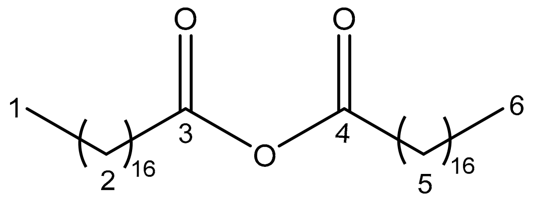

Stearic acid (300 mg, 1.05 mmol) was dissolved in DCM (5 mL) in a round-bottom flask, which was placed in an ice bath to maintain the temperature at 0°C. A solution of DCC (109 mg, 0.53 mmol) in DCM (2 mL) was prepared separately and added dropwise to the cold stearic acid solution with continuous stirring. The reaction mixture was stirred overnight to ensure complete formation of the stearic anhydride. The following day, the reaction mixture was filtered through celite to remove the DCU byproduct formed during the reaction. The solvent was removed under reduced pressure using a rotary evaporator to obtain stearic anhydride as a white crystalline solid. (200.0 mg, 69%). $C_{36}H_{70}O_3$ (550.95 g mol^{-1}). ^{1}H-NMR (400 MHz, $CDCl_3$) δ/ppm: 2.47 – 2.29 (4H, m, H2, H5), 1.68 – 1.60 (4H, m, H2, H5), 1.25 (56H, s, H2, H5), 0.87 (6H, m, H1, H6). ^{13}C-NMR (101 MHz, CDCl3) δ/ppm: 169.76 (C3, C4), 36.12, 35.43, 34.25, 32.07, 31.06, 29.84, 29.80, 29.77, 29.74, 29.71, 29.62, 29.60, 29.54, 29.50, 29.42, 29.33, 29.26, 29.02, 26.54, 25.72, 25.67, 25.62, 24.98, 22.83 (C2, C5), 14.24 (C1, C6).

G2-(PA-NHBoc)$_9$-(Stearate)$_{12}$

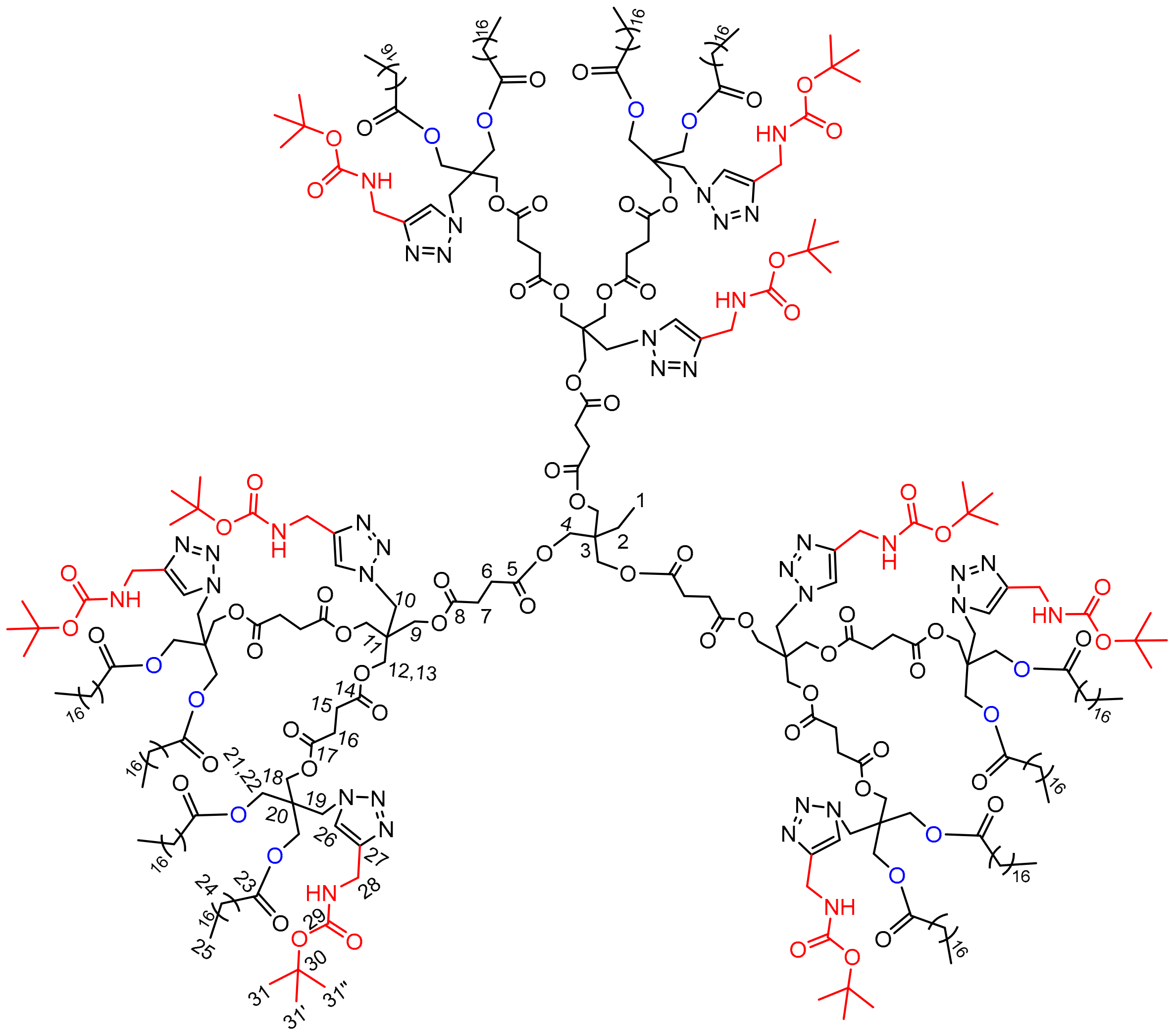

G2-(PA-NHBoc)$_9$-(OH)$_{12}$ (54 mg, 0.015 mmol) was dissolved in DCM (5 mL), in a round-bottom flask. To this solution, DMAP (4.3 mg, 0.035 mmol) and pyridine (70 µL, 0.87 mmol) were added sequentially as bases and catalysts. A solution of stearic anhydride (144 mg, 0.26 mmol) in DCM (5 mL) was added dropwise to the reaction mixture with constant stirring. The reaction was stirred overnight and the reaction progress was monitored by NMR spectroscopy and MALDI-TOF to confirm complete esterification. Upon completion, the reaction mixture was concentrated under reduced pressure and loaded onto a silica gel plug. The crude product was purified using a gradient elution system: initially with 50:50 EtOAc:Hep, followed by 80:20 EtOAc:Hep, then 90:10 EtOAc:MeOH, and finally pure MeOH. Fractions containing the pure G2-(PA-NHBoc)$_9$-(Stearate)$_{12}$ were combined, concentrated under reduced pressure to yield the product as a white solid. (70.0 mg, 70.0 %). $C_{375}H_{656}N_{36}O_{78}$ (6917.55 g mol^{-1}). ^{1}H-NMR (400 MHz, CDCl$_3$) δ/ppm: 7.74 – 7.53 (9H, m, H26), 4.50 (18H, m, H10, H19), 4.34 (18H, m, H28), 4.07 (62H, m, H4, H9, H12, H13, H18, H21, H22), 2.62 (36H, s, H6, H7, H15, H16), 2.30 (24H, m, H24), 1.58 (24H, m, H24), 1.40 (81H, s, H31, H31', H31''), 1.24 (338H, m, H2, H24), 0.86 (39H, m, H1, H25). ^{13}C-NMR (101 MHz, CDCl$_3$) δ/ppm: 173.07, 172.10, 171.72, 171.25 (C5, C8, C14, C17, C23), 156.00 (C29), 145.60 (C27), 123.97 (C26), 79.64 (C30), 64.25 (C4), 63.01, 62.21, 60.49 (C9, C12, C13, C18, C21, C22), 49.86 (C10, C19), 42.90 (C11, C20), 40.85 (C3), 36.05 (C28), 34.12, 33.94, 32.03, 29.82, 29.79, 29.77, 29.63, 29.60, 29.47, 29.42, 29.29, 28.85, 28.50, 28.49, 25.03, 24.94, 22.79, 21.14 (C6, C7,

C15, C16, C24, C31, C31’, C31’’), 14.30 (C25), 14.22 (C25), 7.49 (C1). MALDI: Calc. [$M+K^+$] = 6956.64 Da, Found [$M+K^+$] = 6961.59 Da. SEC (DMF) M_n = 8274.8 g mol^{-1}, M_w= 8471.5 g mol^{-1}, Đ = 1.02.

G2-(PA-NH_3^+)$_3$-(Stearate)$_{12}$

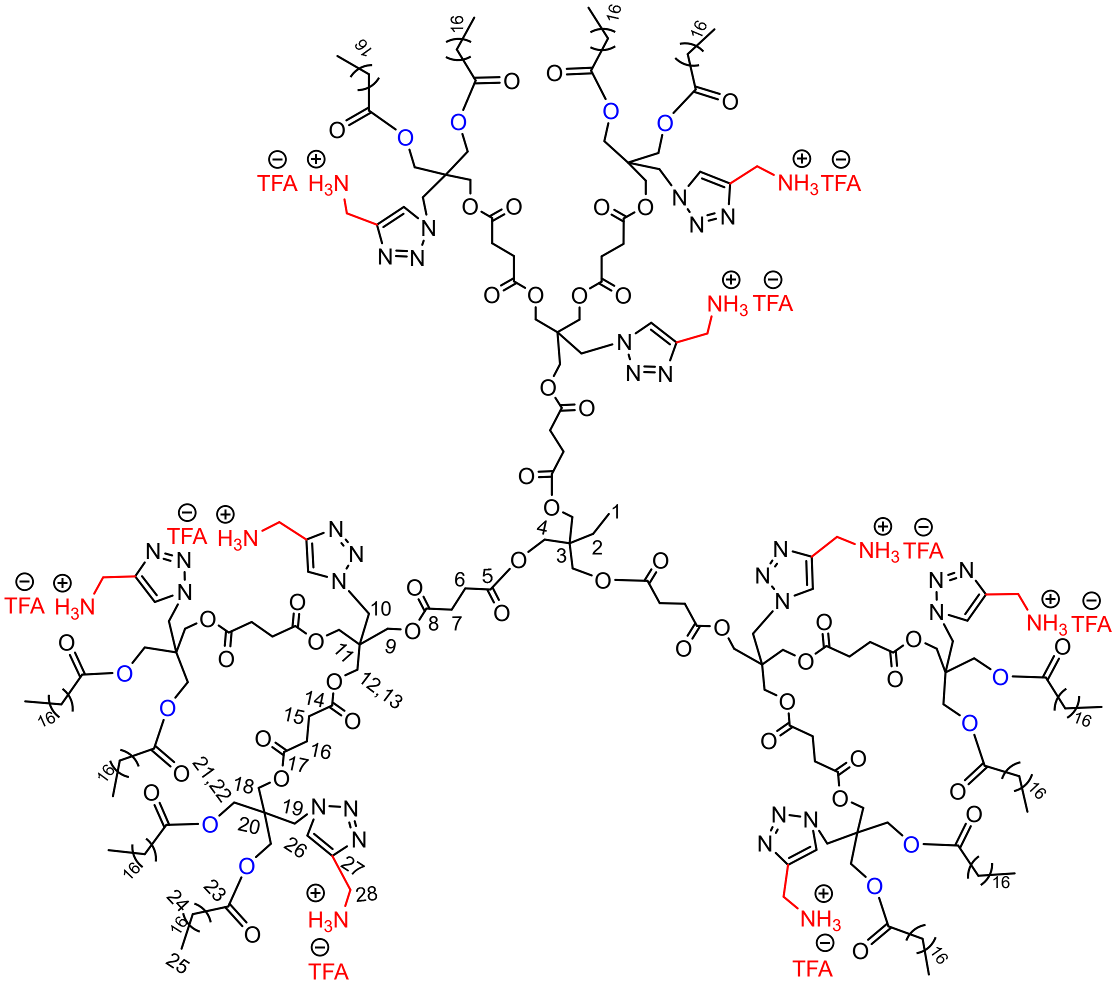

G2-(PA-NHBoc)$_9$-(Stearate)$_{12}$ (38.9 mg, 0.006 mmol) was deprotected in TFA (46 µL, 0.61 mmol) with DCM (46 µL) as co-solvent. The reaction was carried out at room temperature for 4 hours and the reaction progress was monitored by NMR spectroscopy to confirm complete Boc deprotection. Upon reaction completion, TFA and DCM were evaporated and left in the vacuum line overnight. The crude product obtained was purified the following day by precipitation in diethyl ether (3 times) after dissolution in the minimal volume of DCM, with consequent removal of solvent under reduced pressure. The precipitated product was collected by centrifugation, and dried under high vacuum to yield the fully deprotected G2-(PA-NH_3^+)$_9$-(Stearate)$_{12}$ dendrimer as a TFA salt. (39.0 mg, 98%). $C_{348}H_{593}N_{36}O_{78}F_{27}$ (7042.74 g mol^{-1}). ^{1}H-NMR (400 MHz, $CDCl_3$) δ/ppm: 8.07 – 7.94 (9H, m, H26), 4.50 (18H, m, H10, H19), 4.31 – 4.21 (18H, m, H28), 4.12 – 3.87 (60H, m, H4, H9, H12, H13, H18, H21, H22), 2.64 – 2.54 (36H, m, H6, H7, H15, H16), 2.29 (24H, m, H24), 1.55 (24H, m, H24), 1.27 – 1.18 (338H, m, H2, H24), 0.84 (39H, m, H1, H25). ^{13}C-NMR (101 MHz, $CDCl_3$) δ/ppm: 173.38, 172.56, 172.39, 172.17 (C5, C8, C14, C17, C23), 161.63, 161.26 (C=O (TFA)), 140.14 (C27), 126.58 (C26), 117.54, 114.63 (CF_3 (TFA)), 64.38 (C4), 62.74, 61.99 (C9, C12, C13, C18, C21, C22), 50.40, 49.80 (C10, C19), 42.83 (C11, C20), 34.70, 34.28, 34.08, 33.91, 33.28, 32.08, 29.89, 29.88, 29.84, 29.82, 29.80, 29.75, 29.71, 29.60, 29.52, 29.48, 29.40, 29.31, 29.23, 28.78, 25.41, 25.11, 24.95, 24.87, 24.79, 22.84, 22.66 (C6, C7, C15, C16, C24), 14.26 (C25), 7.31 (C1). MALDI: Calc. [$M+Na^+$] = 6048.55 Da, Found [$M+Na^+$] = 6048.49 Da.

Figures

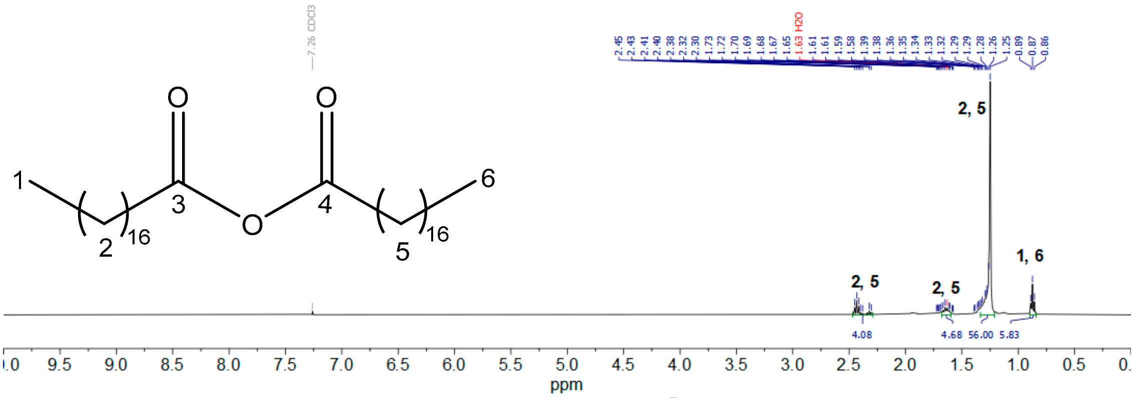

Figure S1. ^{1}H and ^{13}C NMR spectra of Stearic anhydride in $CDCl_3$.

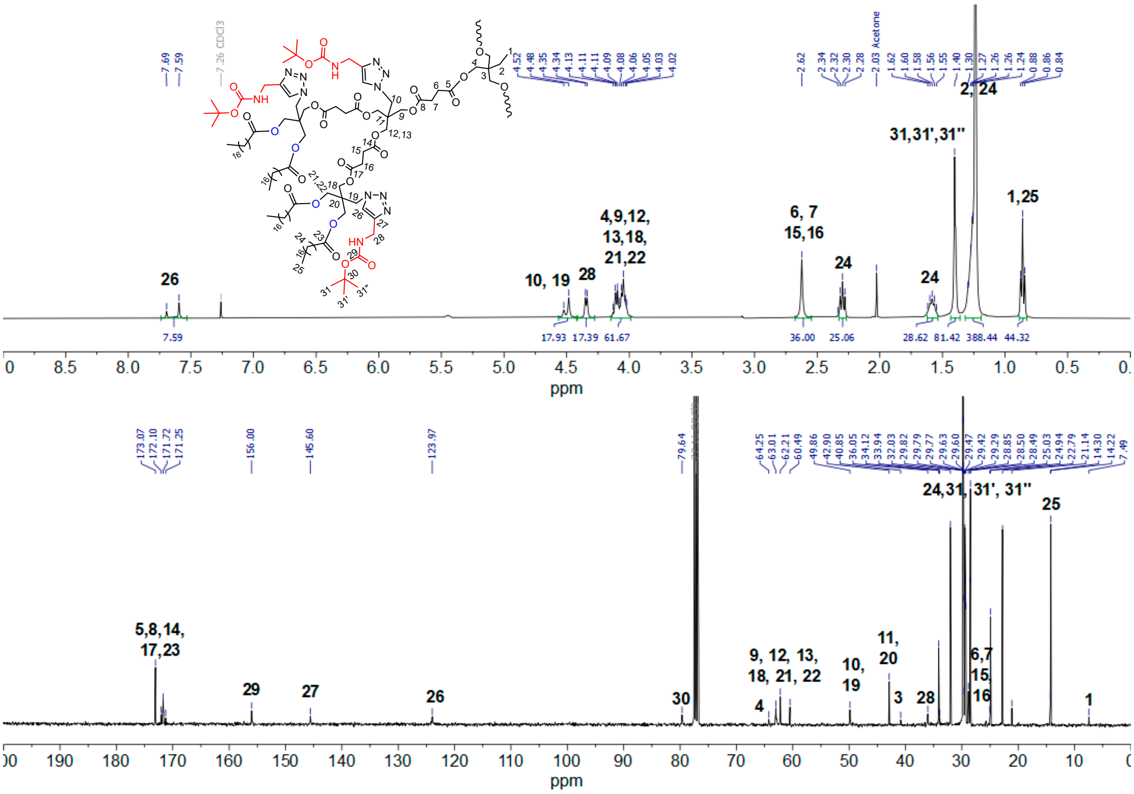

Figure S2. ^{1}H and ^{13}C NMR spectra of G2-(PA-NHBoc)$_9$-(Stearate)$_{12}$ in $CDCl_3$.

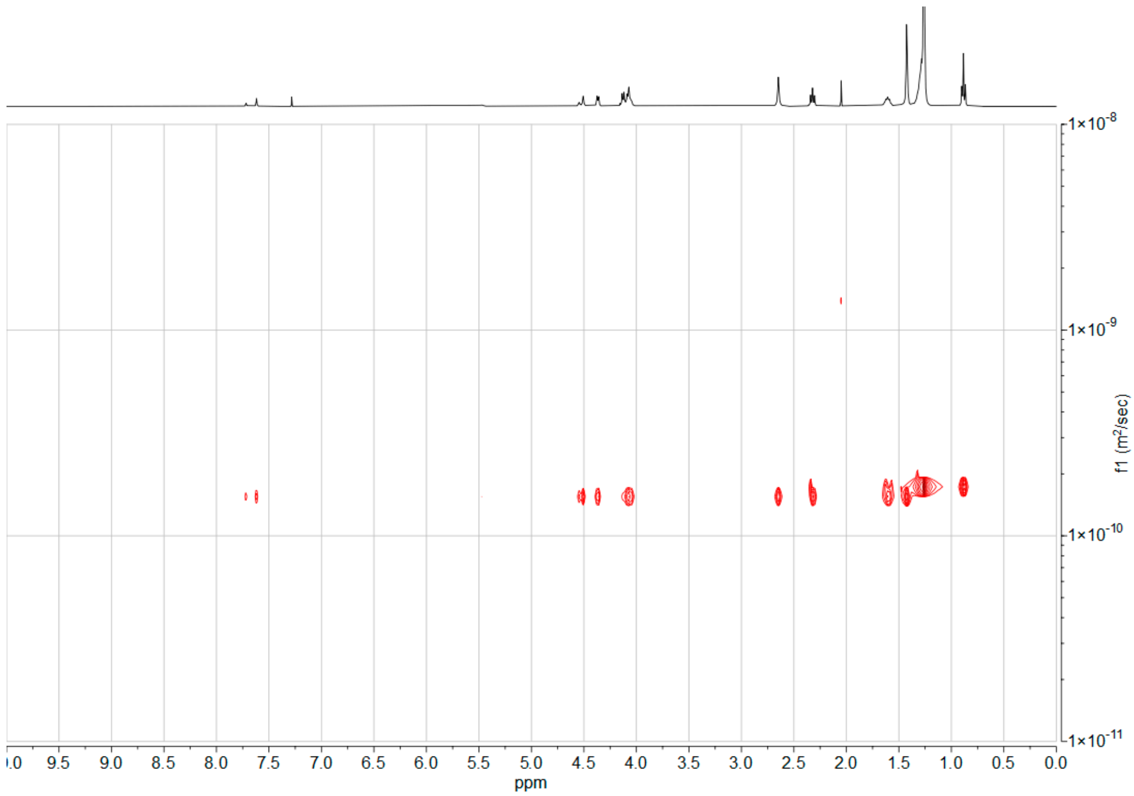

Figure S3. DOSY spectra of G2-(PA-NHBoc)$_9$-(Stearate)$_{12}$ in $CDCl_3$.

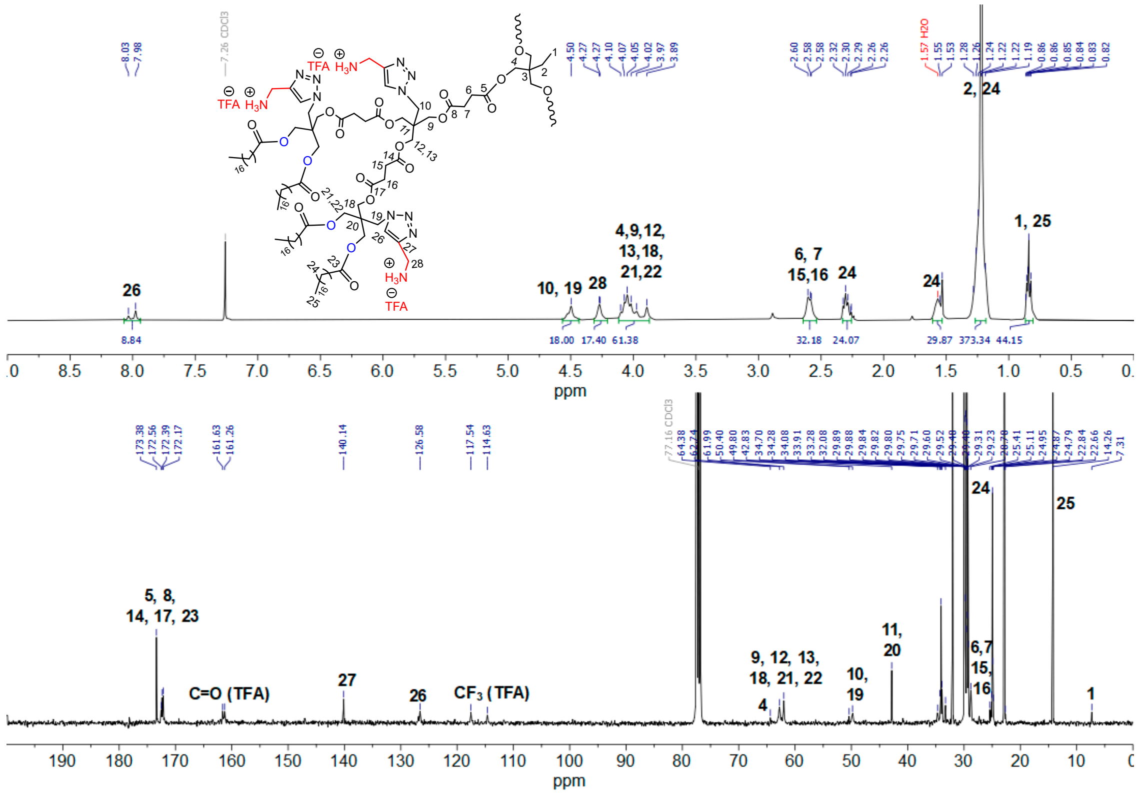

Figure S4. ^1H and ^{13}C NMR spectra of G2-(PA-NH_3^+)$_9$-(Stearate)$_{12}$ in $CDCl_3$.

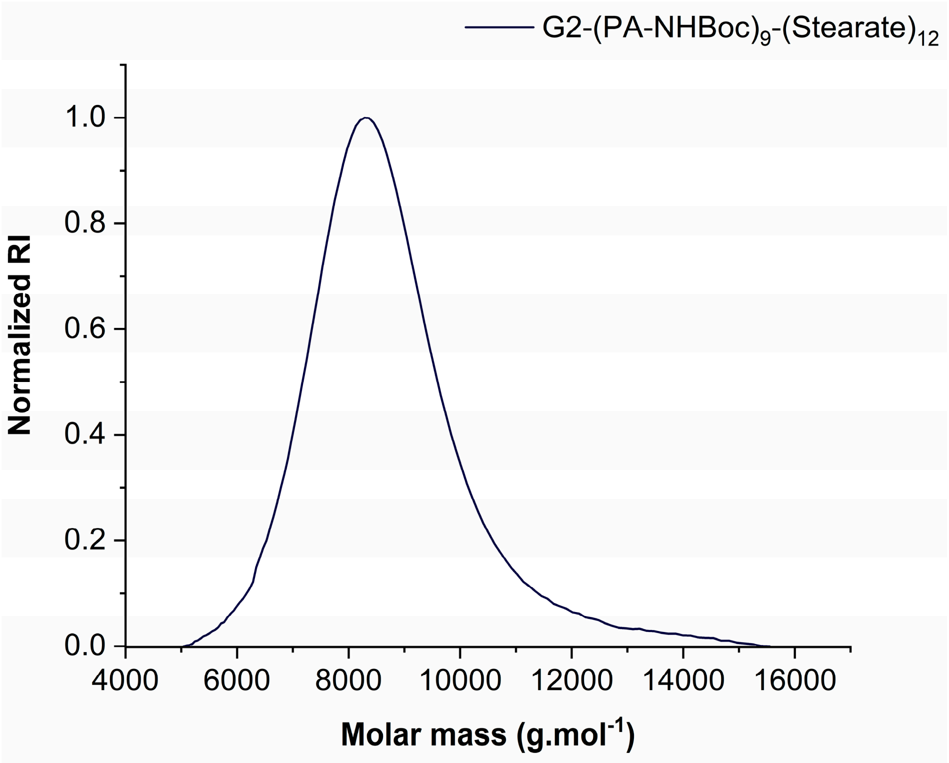

Figure S5. SEC of G2-(PA-NHBoc)$_9$-(Stearate)$_{12}$.

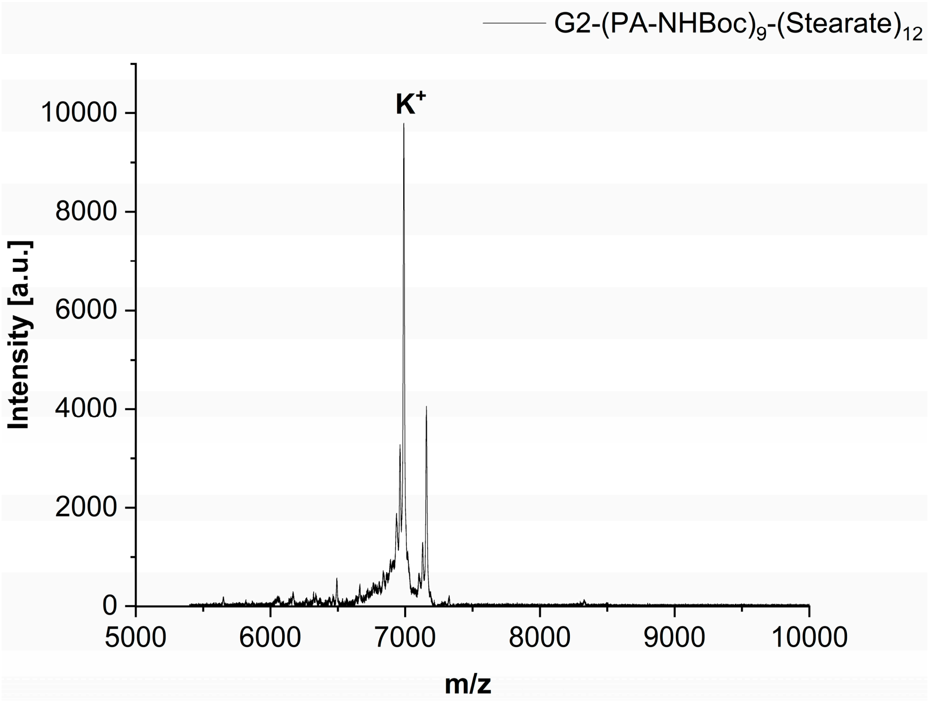

Figure S6. MALDI-TOF spectra of G2-(PA-NHBoc)$_9$-(Stearate)$_{12}$ in DCTB.

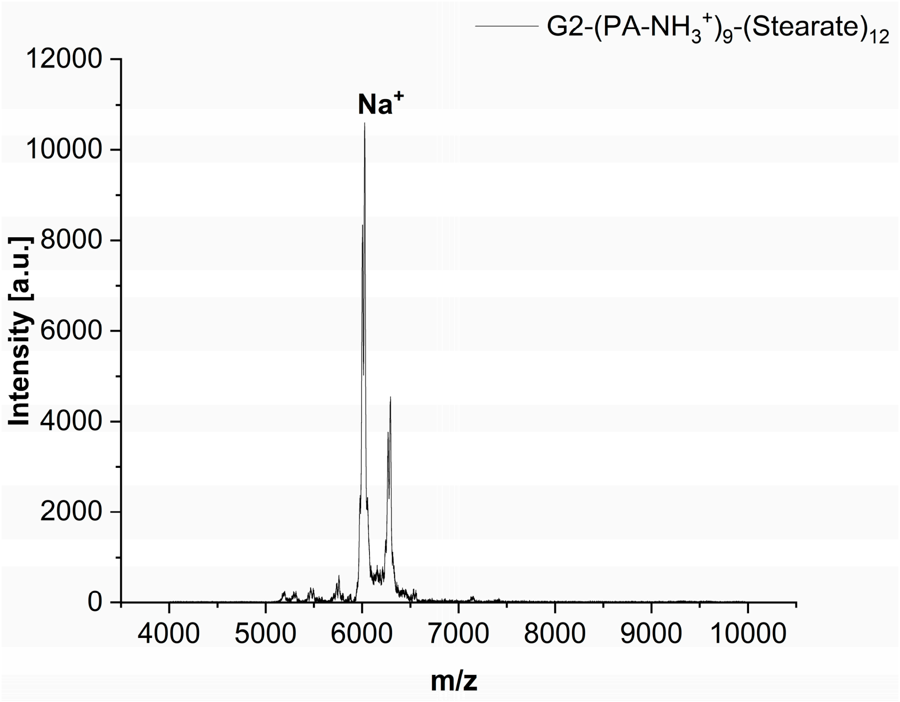

Figure S7. MALDI-TOF spectra of G2-(PA-NH_3^+)$_9$-(Stearate)$_{12}$ in DCTB.

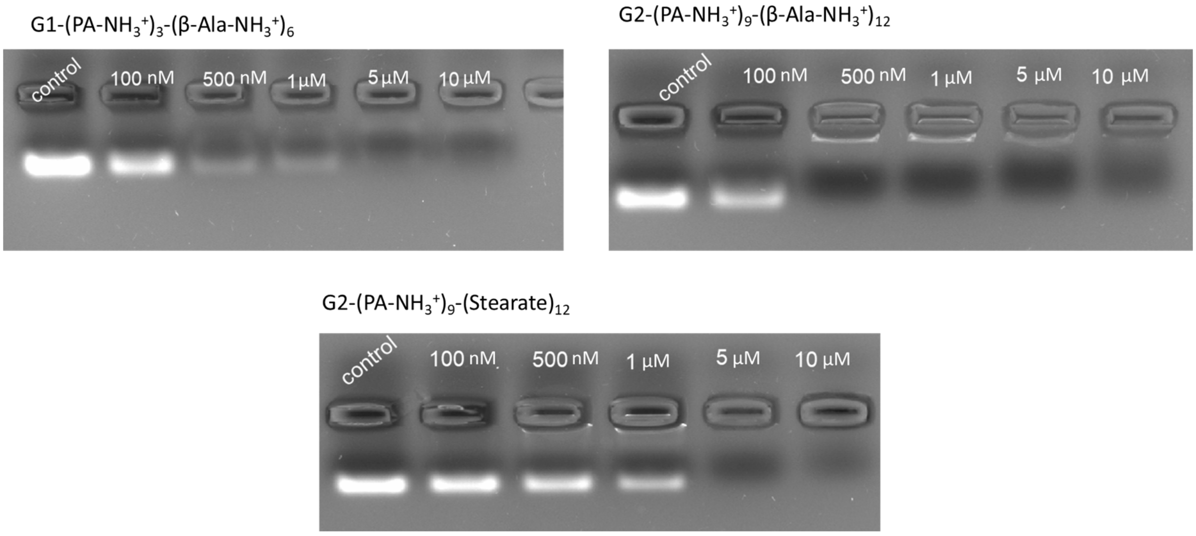

Figure S8. Agarose gel retardation assay of siRNA (100 nM) complexed with G1-(PA-NH_3^+)$_3$-(β-Ala-NH_3^+)$_6$, G2-(PA-NH_3^+)$_9$-(β-Ala-NH_3^+)$_{12}$ and G2-(PA-NH_3^+)$_9$-(Stearate)$_{12}$ from the 2nd family at concentrations ranging from 100 nM to 10 µM. Gel electrophoresis images showing lane 1 as free siRNA (control), with subsequent lanes corresponding to increasing dendrimer concentrations.

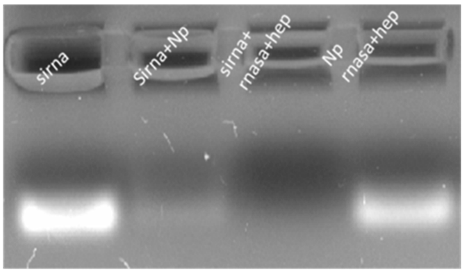

G1-$(PA\text{-}NH_3^+)_3$-$(\beta\text{-}Ala\text{-}NH_3^+)_6$
(1 µM)

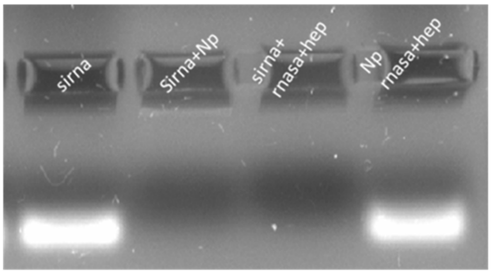

G2-$(PA\text{-}NH_3^+)_9$-$(\beta\text{-}Ala\text{-}NH_3^+)_{12}$
(1 µM)

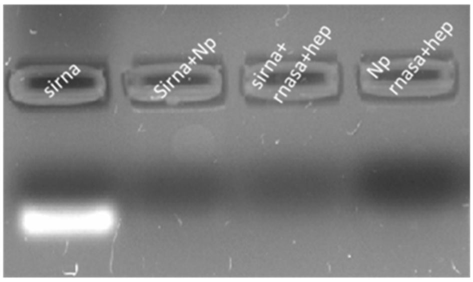

G2-$(PA\text{-}NH_3^+)_9$-$(Stearate)_{12}$
(1 µM)

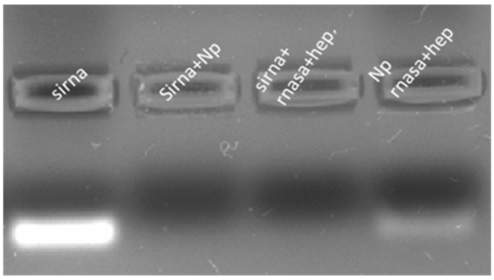

G2-$(PA\text{-}NH_3^+)_9$-$(Stearate)_{12}$
(5 µM)

Figure S9. RNase protection assay of siRNA (100 nM) by the 2nd family cationic dendrimers at 1 µM (G1-$(PA\text{-}NH_3^+)_3$-$(\beta\text{-}Ala\text{-}NH_3^+)_6$, G2-$(PA\text{-}NH_3^+)_9$-$(\beta\text{-}Ala\text{-}NH_3^+)_{12}$) and 5 µM (G2-$(PA\text{-}NH_3^+)_9$-$(Stearate)_{12}$). Dendriplexes were treated with RNase A (0.25 mg/mL) for 30 minutes at 37°C, followed by heparin displacement and agarose gel electrophoresis. Lane assignments: siRNA control, siRNA + nanoparticle, siRNA + RNase A + heparin, and dendrimer control (nanoparticle + RNase A+ heparin).

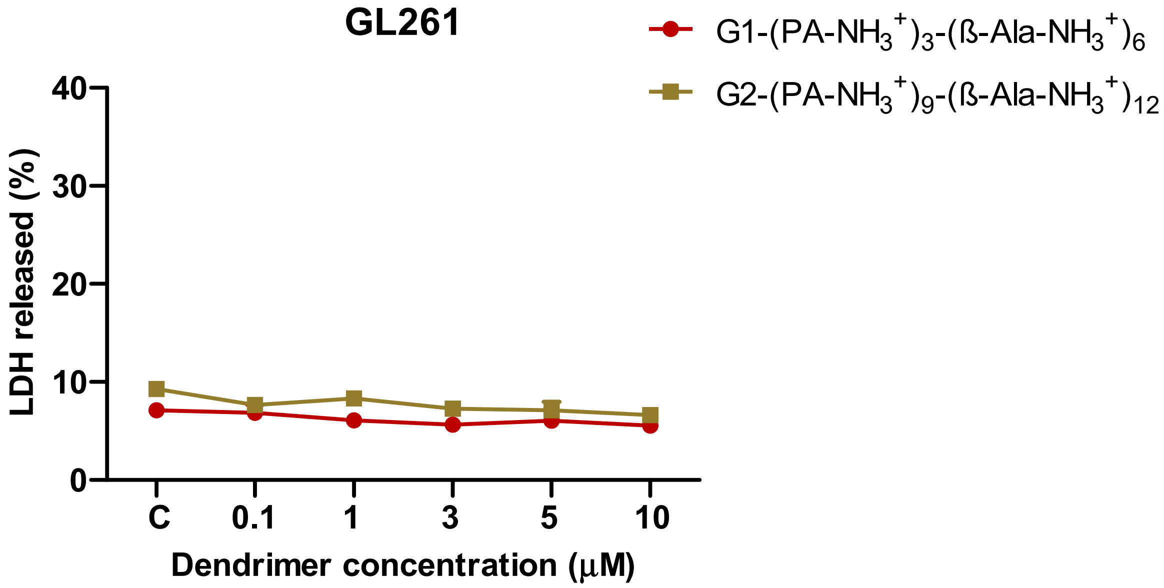

Figure S10. Cytotoxicity of the second family (G1-$(PA\text{-}NH_3^+)_3$-$(\beta\text{-}Ala\text{-}NH_3^+)_6$, G2-$(PA\text{-}NH_3^+)_9$-$(\beta\text{-}Ala\text{-}NH_3^+)_{12}$) cationic heterofunctional dendrimers in GL261 cells following 72h exposure at the indicated concentrations (0.1-10 µM). Data are presented as mean ± s.e.m. (n= 3-4 independent experiments).

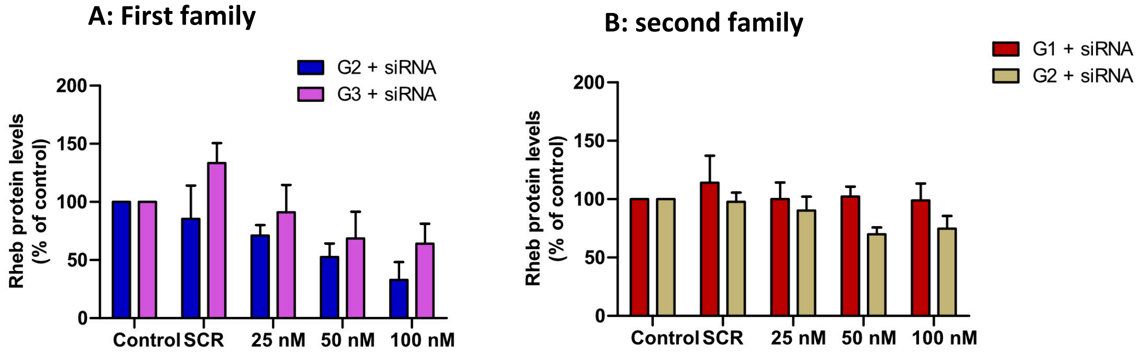

Figure S11. Protein knockdown in T98G cells transfected with dendrimer-siRNA complexes (25-100 nM siRNA) targeting Rheb. Protein levels were quantified after 72 h by Western blot analysis. Data represent mean ± s.e.m (n= 3-4 independent experiments).

References

1. Singh, A.; Sanz del Olmo, N.; Malkoch, M. Heterofunctional Cationic Polyester Dendrimers as Antibacterial Agents: The Role of Internal and External Charges. *Biomacromolecules* **2025**, *26*, 6164-6176.
